# A Triple Mystery of Insidious Organ Failure: Are the Lung, Kidney and Brain All Damaged by the Ageing Pulse?

**DOI:** 10.3390/biomedicines12091969

**Published:** 2024-09-01

**Authors:** Jonathan Stone, Stephen R. Robinson, John Mitrofanis, Daniel M. Johnstone

**Affiliations:** 1 Discipline of Physiology, and Bosch Institute, University of Sydney, Sydney, NSW 2006, Australia; 2 School of Health and Biomedical Sciences, RMIT University, Bundoora, VIC 3083, Australia; stephen.robinson@rmit.edu.au; 3 Fonds de Dotation, Clinatec, Université Grenoble Alpes, 38000 Grenoble, France; john.mitrofanis@univ-grenoble-alpes.fr; 4 School of Biomedical Sciences and Pharmacy, University of Newcastle, Callaghan, NSW 2308, Australia; daniel.johnstone@newcastle.edu.au

**Keywords:** dementia, chronic kidney disease, idiopathic pulmonary fibrosis, capillary haemorrhage, hypertension, healthy ageing

## Abstract

This review explores the hypothesis that dementia in several forms, chronic kidney disease and idiopathic pulmonary fibrosis have a common cause in pulse-induced capillary haemorrhage. All three conditions are age-related and characterised by insidious onset, uncertainty about their cause, exacerbation by hypertension, resistance to treatment and the relentlessness of their progression. We argue that the three conditions are the clinical outcomes of damage caused by pulse-induced haemorrhage from capillaries. The damage, first detectable in mid-life, creates first mild and then severe symptoms of cognitive, renal and pulmonary dysfunction. We also review evidence that in all three organs there has developed, by young adulthood, a reserve of tissue that enables them to function well, despite the ‘heartbeat by heartbeat’ damage that accumulates from early mid-life; and that it is when that reserve is exhausted, typically in late age, that symptoms of organ failure emerge and progress. If this common cause can be established, a step will have been taken towards the understanding, treatment and delay of three conditions that have their beginnings in every individual and that, in those who survive other causes of death, become lethal in late age.

## 1. Introduction

This essay addresses a reality of ageing that has become increasingly clear as longevity has increased in many societies: that three organs—the brain, kidney and lung—can fail, gradually but fatally, in later life, without clear cause. The results of their failure—dementia, chronic kidney disease (CKD) and idiopathic pulmonary fibrosis (IPF)—are often lethal and difficult to treat. We have argued elsewhere that the pathological hallmarks of dementia (senile plaques) accumulate in every person with age, marking sites of capillary haemorrhage made increasingly frequent by the ageing pulse [1,2] and slowly destroying the brain in a seemingly healthy body. Here, we explore whether the kidney and lung share this cumulative pattern of pulse-induced pathology, leading insidiously from minor, statistically normal pathology to lethal breakdown of the organ.

The insight that the ageing pulse destroys the ageing brain deserves context. It was proposed by vascular biologists [3,4,5,6,7] who understood the stiffening with age of the walls of the aorta and of the great distributing arteries (femoral, brachial, cerebral), and the effect of that stiffening on systolic and pulse pressures. Neuroscience-based investigators, searching for the cause of age-related degenerations of the brain and of the dementias they caused, understood that thrombosis of or haemorrhage from large cerebral vessels causes clinically evident episodes (strokes) and a cognitive loss termed vascular dementia. They were concerned with age-related dementias like sporadic Alzheimer’s, whose sufferers had no history of stroke or cerebral disease. The most influential concept to emerge from neuropathology-based studies is that the ageing brain, for still enigmatic reasons, starts to express self-toxic proteins (the most notorious being the peptide Aβ), which start a cascade of amyloid toxicity [8,9,10]. The insights of the vascular biologists were integrated with the neuropathology of dementias by investigators who proposed that Aβ^+^ plaques form at the sites of and downstream from capillary haemorrhages too small to cause symptoms [2,11,12] and who, in an additional step, argued that it is the ageing pulse that causes the capillary haemorrhages [13]. This review argues that, by causing such haemorrhages, the ageing pulse destroys the kidney and lung, as well as the brain.

Why are these three organs affected, with such different functions? A case for considering a common pathology is presented below, beginning with two sets of observations: (1) the fact that many features of the failures of these organs during late age are common to the three; and (2) the view, mentioned above, that pulse-induced capillary haemorrhage is destructive to the brain [1,2,12,14,15,16]. With those two starting points, the question becomes whether ageing-pulse-induced capillary haemorrhage (APICH) is also significant in the pathology of chronic kidney disease and of idiopathic pulmonary fibrosis. For an answer to that question, we next trace the available data relevant to the two starting points, and then bring together whatever evidence we could find, from the peer-reviewed literature, of APICH in CKD and IPF. Finally, implications of these observations for the understanding of human ageing are discussed.

### 1.1. Starting Point #1: Common Features of the Pathogenesis of Dementia, CKD, and IPF

#### 1.1.1. Common Clinical Features

Dementia, CKD and IPF are degenerative conditions, all progressive and, so far, difficult-to-impossible to stop or reverse; lethal without the radical intervention of dialysis (for the kidney) or transplants (for the kidney and lung); age-related and of unknown—or at best debated—cause. As would be expected if they are all caused by a common factor, dementia, CKD and IPF are commonly co-morbidities in older people, so much so that consideration has been given to whether CKD [17] or IPF is a ‘risk factor’ for cognitive loss. In 2009, for example, Ito and colleagues [18] proposed a ‘strain vessel hypothesis’ to explain the close association between albuminuria (as a measure of kidney failure) and stroke or cardiovascular disease; Kelly and colleagues [19] extended the idea to include ‘inflammatory cross-talk’ [20] to explain why sufferers of CKD have a 2- to 3-fold greater risk of developing dementia. For the lung, evidence seems clear that several lung conditions suffered in mid-life are associated with an increased risk of dementia [21,22], although few cohort studies that test for an association between IPF and subsequent dementia have been reported. Bors [23] studied cognitive function (speed of recognition tasks) in patients with IPF but did not extend observations to the risk of later development of dementia, possibly because the course of IPF is, among these three conditions, relatively rapid.

#### 1.1.2. Pathology: Shrinkage and Fibrosis/Gliosis

At the whole-organ level, all three organs shrink slowly with age. In the brain, shrinkage of 0.5–1.0% p.a. is considered normal after early mid-life, is most marked in the cortical grey matter [24], which is essential for cognition, and is associated with small reductions in cognitive performance, also considered normal. FJell and colleagues concluded, for example [25], that …reductions in specific abilities are to a substantial degree mediated by (normal) neuroanatomical changes. Piras and colleagues [26] noted that maximal kidney size is closely related to birthweight and declines (from the fourth decade of life in men, earlier in women), the decline accelerating with age. The ‘size’ of the lung is often measured as its vital capacity, the largest breath the individual can take and then exhale. In each individual, vital capacity increases sharply during childhood but, after ~25 years of age, decreases slowly during normal/healthy ageing [27], falling to 70% of young adult values in advanced age [28], so, again, ~0.5–1.0% p.a.

At the light microscope/cellular level in all three conditions, the organs show sites of degeneration of functional tissue and the appearance of non-functional (gliotic, fibrotic) tissue at those sites. In the brain, the degeneration is seen in small (<100 µm dia.) patches of neuronal death, gliosis, proteinopathies and signs of capillary haemorrhage [2,11]. These patches were described by Alzheimer [29] (who called them *Herde* (G.—foci)); in English, they are termed senile plaques and have long been considered one of the ‘hallmarks’ of Alzheimer’s dementia (see, for example, https://alzheimer.ca/en/about-dementia/what-alzheimers-disease/how-alzheimers-disease-changes-brain (accessed on 1 July 2024)). The idea that dementia might be a primarily ‘capillary dementia’ was proposed 35 years ago by Scheibel and colleagues [30]. De la Torre [31] developed their proposal, describing Alzheimer’s dementia as a ‘vasocognopathy’ caused by capillary leakage. The idea was further developed by investigators who, because of evidence of blood-specific proteins (fibrinogen, von Willebrandt’s factor, haemosiderin) and blood cells in the neuropil of end-stage Alzheimer brains, argued that the key capillary dysfunction in age-related dementias like Alzheimer’s may be haemorrhage rather than leakage [2,11].

In the kidney, in CKD, the functional tissue that dies includes the specialised capillary structure known as the glomerulus (in the cortex) and the peritubular capillary network (in the medulla), as well as the tubular structures they supply, including the nephron. The hallmarks of CKD include *tubulointerstitial fibrosis*, *tubular atrophy* and *peritubular capillary rarefaction* [20] at the sites of functional cell loss. Although ‘rarefaction’ of capillaries is clearly described [32], descriptions comparable to the those available for the brain, in which tests of haemorrhage (haemosiderin, blood-specific proteins at the sites of degeneration) had been applied, could not be found in the literature. In the lung, in IPF, the functional tissue that dies includes the endothelium of the alveolar capillaries, and the hallmark pathology is the fibrosis found at the sites of alveolar death. The areas of fibrosis have few blood vessels and are often surrounded by abnormal vessels [33,34], also considered a ‘hallmark’ pathology [35]. As in the kidney, capillary haemorrhage has apparently not been directly investigated in the IPF lung.

#### 1.1.3. All Three Are Highly Vascular

The vascularity of these three organs is the stuff of textbooks. In the body at rest, the brain (~2% of body weight) requires 20% (~1 L/min) of the resting systemic cardiac output [36], and the kidneys (together ~0.4% of body weight) require their own 20%. Thus, the brain and kidney are two to three orders of magnitude more vascular by weight than ‘the rest of the body at rest’, with one major exception: the lungs receive a total cardiac output of 5 L/m in a separate pulmonary circulation, operating in series with the systemic. (Strictly, the lungs receive more than the total cardiac output. They receive 100% of cardiac output from the right ventricle, and a small volume, perhaps never measured, from small branches of the systemic (left ventricle) circulation, via the bronchial arteries, which supply the structural (non-aerating) tissues of the lung; see [37].)

The three organs are so vascular for distinctive reasons. The brain requires high blood flow to support the energy-intensive ion pumps that maintain the transmembrane voltage gradients that subserve neural activity. Correspondingly, the brain regulates its blood flow according to metabolic needs—the supply of oxygen and glucose and the removal of CO_2_. Further, the brain can regulate its blood flow in a regional way; changes in mental/cerebral activity (sensory or motor or emotional or puzzle-solving) produce regional changes in blood flow. These local changes, studied in vivo, have yielded much understanding of brain circuitry.

By contrast, blood flow to the kidney is regulated to serve the organ’s waste-removal functions, by a combination of vascular and tubular mechanisms, novel to the kidney, which provides for high autoregulatory efficiency that maintains renal blood flow and glomerular filtration rate, stabilises sodium excretion, and buffers the transmission of renal perfusion pressure to sensitive glomerular capillaries, thereby protecting against hypertensive barotrauma [38]. In the kidney, then, regulation maintains blood flow and excretory function in the face of variations in factors such as systemic blood pressure or blood oxygen levels. In a further contrast, the pulmonary circulation is regulated by neural and humoral mechanisms [37] that optimise the oxygenation of blood and the removal of carbon dioxide from the blood, again maintaining the primary function of the organ. (The lungs evolved in fish as ventral ‘buds’ from the gastrointestinal system. In transitional forms like the lungfish, the lungs were a new organ in the vertebrate body plan and might have evolved to receive their blood from the systemic circulation, as do the respiratory organs of fish (the gills). In fact, the evolution of the lungs included a longitudinal splitting of the heart into left and right pumps, one for the lungs, the other for the rest of the body. The great arteries and veins evolved connections so that the left and right circulations operated in series, necessarily with identical stroke volumes. As a consequence, the lungs are perfused by the same volume as all of the rest of the body (~5 L/min). They are by far the most vascular organ of the body, in terms of both the absolute blood flow through them and blood flow per unit weight). Among these considerable differences in regulation, however, a common factor remains: all three organs are highly vascular, arguably the most vascular organs of the body

#### 1.1.4. Low Resistance, High Pulsatility

A corollary of the high blood flows that perfuse the brain, kidney and lung is that their vascular beds are of low resistance/high compliance.

Compliance of any circulation can be calculated as follows:

**C = V/P** L/mm Hg

where C is compliance, V is the volume of blood flowing through the system (organ or limb) and P is pulse pressure (the difference between systolic and diastolic pressures). V is 5-fold higher for the lungs than for the brain or kidneys, and in addition, pulse pressure is ~4-fold less.

Compliance is ~20-fold greater in the lungs than in either the brain or kidneys and, in all three, compliance is much greater than in tissues like resting muscle or skin. One result is that the pulse penetrates into the capillary beds of all three organs and can be detected in their veins [39,40]. Further, in the living animal, the brain and kidney pulsate visibly [6,41]; pulsations of the lung have been described, but mainly in abnormal conditions such as atelectasis [42]. The capillary beds of the three organs are thus exposed to what Carlström and colleagues termed ‘hypertensive barotrauma’: the impact of the ageing pulse [38]. The pulsatile heart is a mechanism evolved in vertebrates to drive the circulation, but in late life—at least in humans, given our longevity—the pulse has the potential to damage low-vascular-resistance organs.

#### 1.1.5. Relation to Age: The Reassuring Normality of Lethal Disease

All three organs have been shown to reach their functional maxima in the third and fourth decades of life, after which function declines slowly. This slow decline is seen in every individual and so is statistically normal. For example:

CKD becomes more common with increasing age. After the age of 40, kidney filtration begins to fall by approximately 1% per year. (https://nccd.cdc.gov/ckd/FactorsOfInterest.aspx?type=Age#:~:text=However%2C%20CKD%20becomes%20more%20common,blood%20pressure%2C%20and%20heart%20disea) (accessed on 1 July 2024).

Loss of kidney function with age is thus ubiquitous. Should we be concerned about it in ourselves? The answer seems to be yes and no; ‘no’ because what is ubiquitous is statistically normal, and the individual has not made bad choices beyond surviving into late age; and ‘yes’ because loss of kidney function can continue, just with age, to levels so low that it becomes lethal. Again:

Early-stage kidney disease is so common amongst older people that some experts argue that it shouldn’t be considered an illness, but just part of normal ‘wear and tear’ ageing. (https://www.kidneyresearchuk.org/kidney-health-information/about-kidney-disease/am-i-at-risk/age-and-kidney-disease/#:~:text=Chronic%20kidney%20disease&text=Early%2Dstage%20kidney%20disease%20is,'wear%20and%20tear'%20ageing) (accessed on 1 July 2024).

And similarly for the lung:

After about the age of 35, it is normal for your lung function to decline gradually as you age. This can make breathing slightly more difficult as you get older. (The American Lung Association: https://www.lung.org/lung-health-diseases/how-lungs-work/lung-capacity-and-aging#:~:text=After%20about%20the%20age%20of,to%20your%20doctor%20right%20away) (accessed on 1 July 2024).

The brain also shows highly reproducible functional declines with age. For instance, task-switching performance, inhibitory control and reaction time all improve until the mid-twenties and thereafter performance relentlessly declines [43,44]. The impairment of cognition in mid-life begins with subtle declines and can eventually progress to the tragedy of dementia.

The point of this section is to note that, in all three organs, the statistically normal, slow degeneration of mid-life continues into old age, becoming lethal. But the pathologies do not change qualitatively; lethal disease of old age seems to be continuous with the normal decline of mid-life.

#### 1.1.6. Hypertension Is a Risk Factor for All Three

Epidemiological studies have provided evidence that hypertension is a risk factor for dementia [6,7,45,46], CKD [6,47] and other forms of kidney damage [48] and for IPF [49]. Further, evidence seems clear that hypertension is a risk factor for silent microbleeds in the brain [50,51] and is a major contributor to end-stage renal disease (including CKD), with CKD sufferers vulnerable to even modest rises in blood pressure [52]. Wan and colleagues [53] have discussed a ‘vascular-renal’ connection that makes patients with severe hypertension vulnerable to acute kidney failure. We have been unable, however, to find evidence, or even speculation, in the literature that hypertension causes or contributes specifically to capillary haemorrhage in the kidney.

In the lung, hypertension has for some time been considered to be ‘associated with’ IPF (https://www.mayoclinic.org/diseases-conditions/pulmonary-fibrosis/symptoms-causes/syc-20353690) (accessed on 1 July 2024), but the literature is not clear on whether IPF causes the hypertension or vice versa, or possibly both. Hypertension is considered an important cause of diffuse alveolar haemorrhage [54,55,56], but whether it is reasonable to extrapolate from diffuse haemorrhage from alveolar capillaries to the symptom-free bleeding of one capillary at a time, such as occurs in the ageing brain, and that we posit underlies IPF, needs further consideration (below).

#### 1.1.7. Reserve Tissue, Clinical Threshold

We have noted above that the brain, kidney and lungs shrink from early mid-life (mid-30s) by ≤0.5% p.a. without clinical symptoms; more dramatically, humans can live active lives with one kidney (https://www.kidney.org/atoz/content/onekidney#:~:text=A%20solitary%20functioning%20kidney%20means,healthy%20lives%20with%20one%20kidney) (accessed on 1 July 2024) or one lung (https://www.hopkinsmedicine.org/health/treatment-tests-and-therapies/pneumonectomy#:~:text=Most%20people%20can%20get%20by,the%20side%20of%20your%20body) (accessed on 1 July 2024). In humans with normal cognition, Scahill and colleagues [57] reported an annual loss of 0.3% p.a. of brain volume for adults in mid-life, accelerating to 0.5% in octogenarians; similarly, Takao and colleagues [58] reported an average annual loss of 0.23% of brain volume, increasing with age. Overall, these figures suggest that the brain typically shrinks by 15 to 30% from mid- to late life, with cognition remaining clinically normal.

These observations suggest that all three organs develop, during growth to young adulthood, a reserve of deployable tissue. During the decades between mid-life, when the organs start to shrink, and late life, when symptoms of organ failure start to be clinically significant, it seems that this reserve is seamlessly deployed to maintain close-to-normal performance, and that clinical disease is diagnosed when that reserve is exhausted.

#### 1.1.8. Relentless Progression

One further feature common to dementia, CKD and IPF is that, once diagnosed, they progress to lethality. That harsh reality is softened for the kidney and lung by the possibility of organ transplant and for the kidney by dialysis. The progression of dementias has been attributed to the continued beating of the heart, and thus to APICH [15]. The point has been made previously that this ‘heartbeat after heartbeat’ stress on the endothelium of vessels is likely to affect all tissues [47,59], and therefore to affect the kidney and lung, as well as the brain. Below, we review available evidence of capillary haemorrhage for the three organs considered here.

#### 1.1.9. The Pathology of Normal Ageing Is Also the Pathology of the Lethal Condition

Perhaps the feature of the pathologies of dementia, CKD and IPF that seems to provide strongest support for the APICH hypothesis is that, in each condition, the pathology of normal ageing is also the pathology of the lethal disease. In the brain, senile plaques have been observed, in very small numbers, in young adults; they become more frequent with age [2] and are so profuse in the end-stage Alzheimer brain that they regarded as the ‘hallmark’ of Alzheimer’s dementia [10].

In the kidney, Glassock and colleagues surveyed a cohort of over 1000 kidneys in vivo, scanned because they had been offered as transplants. They reported that ‘sclerotic’ changes and larger ‘cysts’ were detectable in a minority of young (third decade) adults and became more frequent with age, detected in over half the kidneys scanned in healthy adults aged 60–70 years. These are the pathologies that are associated with CKD, sites at which functional kidney structures (glomeruli, nephrons, tubules) have died, vessels have thinned and fibrosis is prominent.

We were unable to identify a survey of fibrosis in the normal, healthy lung, comparable to the survey of the living kidney just discussed. The clearest evidence of cumulative lung pathology in healthy adults is the slow decline, discussed above, of vital capacity, which begins in mid-life and extends into late age. This loss seems to be continuous from mid-life to the dyspnoea of age and from there to IPF; but a survey of ‘normal’ pathology in the ageing lung is needed to test this idea.

Thus, in each of the three organs, normal ageing and lethal disease seem to lie along a continuum of the same pathology, considered normal in the healthy young and lethal in the ailing aged. The suggestion that the same pathology can be both normal and lethal seems illogical, but this is how the issue is currently discussed. This normalcy of lethal pathology, at low levels in healthy mid-life, has led some investigators to argue, for example, that cognitively normal adults in whom some senile plaques are present must ‘have’ Alzheimer’s disease, but in a ‘prodromal’ stage [60]. The problem lies in the words ‘normal’ and ‘pathological’. Their meanings seem clearly different and opposite. But lethal pathology appears in the brain, kidney or lung without troubling symptoms (so is a ‘part of normal ageing’) and appears in probably everybody (so is ‘statistically normal’). The ordinary meanings of the two words have come to overlap, and that creates a logical tension: the normal includes pathology, and the pathological can be normal.

Without trying to resolve that tension, it seems reasonable to suggest that the tension can be relaxed by accepting that dementia, CKD and IPF are the end stages of age-related degenerations found in us all. And that acceptance opens the way to consider that their pathogenesis may have a common feature, which we suggest is APICH.

### 1.2. Starting Point #2: Evidence of APICH in the Ageing Brain

The evidence that the pathogenesis of several dementias (pugilistica, chronic traumatic encephalopathy, traumatic brain injury, Alzheimer’s) involves APICH is substantial. We have proposed the idea in several places [1,2,12,15,16,61]), building on the work of many others. In brief summary, Alzheimer [29,62] and his influential mentor Kraepelin [63] both reported vessel breakdown in the post-dementia brain tissue they examined. They did not, however, focus on the vessel pathology, stressing that the condition seemed distinct from the dementias then known, including vascular dementia. From the 1990s through the 2010s, reports appeared (we were particularly influenced by Miyakawa [64,65,66], de la Torre [67], Kumar-Singh [68,69] and Cullen [2,11]) that the senile plaques that form in the ageing human brain, and in mouse models of this ageing, form adjacent to or surrounding capillaries. This seemed to suggest [2,12,70] that senile plaques form at the sites of capillary bleeding. To test this, we were able to reproduce plaque pathology in considerable detail by causing bleeding in the healthy cortex of a wild-type rodent [71]. This idea—that capillary haemorrhage causes the pathology seen as senile plaques—gave support to the ideas of Bateman [3], Henry-Fuegas [4,72,73], O’Rourke [5] and Thorin [47] that the ageing pulse causes an ‘encephalopathy’. The two lines of evidence of the cause of age-related dementia were brought together in [14]. Other investigators, for example [74], have noted that cerebral microhaemorrhages may cause cognitive decline without discussing a link to the pulse. Also without drawing a link to the pulse, Viengkhou and colleagues [75] have reported that the rare, genetic dementia known as Aicardi-Goutières syndrome, caused by the overproduction of interferon-α, is mediated by the toxicity of high interferon-α levels for the endothelial cells of cerebral capillaries. Again, dysfunction of the capillary bed results in a devastating dementia. As a further point, noted above, epidemiological evidence indicates that age-related hypertension, whether assessed as pulse wave velocity (a measure of arterial stiffening) [6,7,45,76,77], as pulse pressure [46] or as the augmentation index (the summing of direct and reflected pulse waves) [77], is associated with a raised risk of cognitive loss and dementia, reinforcing the idea that the pathology of this dementia has a vascular component.

The concept of APICH was extended to chronic traumatic encephalopathy, dementia pugilistica and traumatic brain injury (dementia following external trauma to the head from accidents or combat) by [15] and the toxic impact of even small haemorrhages on neural tissue was re-examined in [16]. Finally on this point, the neuroprotective roles of Aβ were demonstrated by Puzzo [78,79,80], Chuang [81] and Robinson [82,83,84]. These roles include neurotrophic effects, the detoxification of haemoglobin and the sequestering of opportunistic pathogens [85,86,87]. These findings strengthen the idea, first argued by Smith and colleagues [88], that Aβ is not a toxin, but a protective, first-responder molecule, found reliably in senile plaques (the sites of capillary haemorrhages) because it is ‘the fireman, not the arsonist’.

## 2. Evidence That the Kidney and Lung Are Also Damaged by APICH

**Figure 1:**
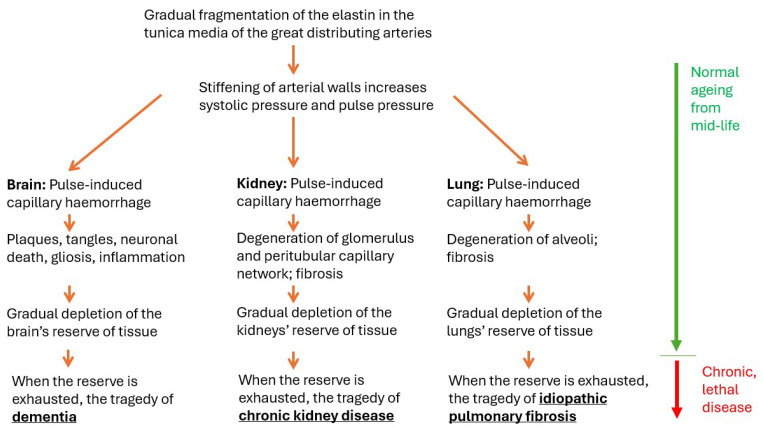
A schema of the APICH (ageing-pulse-induced capillary haemorrhage) hypothesis of the causes of the several dementias considered here, as well as of CKD and of IPF. In the brain, kidney and lung, the loss of function begins insidiously in early mid-life and progresses slowly and normally with age, without crisis or more than mild symptoms. The loss of function is driven—we argue—by the impact of the ageing pulse on the capillary beds of these three very vascular organs, in which pulsatile damage causes the capillaries to haemorrhage. All three organs develop a reserve of deployable tissue during childhood, which can be redeployed to maintain close-to-normal functional levels, despite the damage caused by the small haemorrhages. But when the reserves are depleted, typically in late age, symptoms of cerebral, renal and pulmonary dysfunction appear and become chronic, progressing to lethality. For the kidney and lung, this harsh inevitability is softened by the availability of transplants, and for the kidney, by the availability of dialysis.

**Figure 2:**
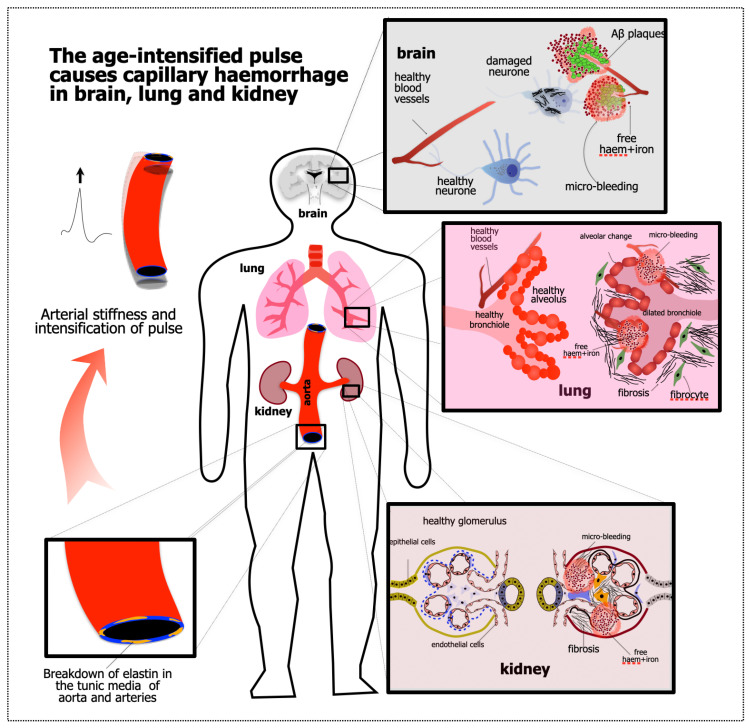
A more diagrammatic/anatomical outline of the APICH hypothesis. The pathology begins away from either the brain, kidney or lung; it begins in the aorta and the great distributing arteries of the body, in which the elastin in their tunica media fragments with age. The increased stiffness of these vessels increases the sharpness of the pulse, which damages capillaries of all three organs. In the brain, capillary haemorrhage leads (according to this hypothesis) to the pathology of proteinopathies and plaque formation; in the kidney, to the death of glomeruli and tubules and to the fibrosis of the sites of such death; and in the lung, to the fibrosis of damaged alveoli.

The evidence for APICH in dementia is summarised immediately above (Starting Point 2). What evidence is there that APICH also occurs in the ageing kidney and lung? The evidence supporting our APICH hypothesis is summarised in Figure 1 and Figure 2.

### 2.1. Kidney

The slow shrinkage and degeneration of the kidney in normal life are mentioned above. Evidence of capillary involvement in this degeneration can be found in reports of several forms of kidney disease. Renal vasculitis, in various forms [89], involves the (usually autoimmune) destruction of glomeruli and results in haematuria, dying off of the glomerular capillaries, fibrosis and the formation of cysts [90], a pathology similar to the end-stage pathology of CKD. Kida and colleagues [91] noted that peritubular capillaries undergo apoptosis during CKD, leading to peritubular rarefaction (and) interstitial fibrosis … hallmarks of CKD. The vascular changes in CKD include an antiangiogenic environment ……. deprivation of EC survival factors, increased production of vascular growth inhibitors, malfunction of ECs, dysfunction of endothelial progenitor cells, and loss of EC integrity via pericyte detachment from the vasculature.

Haematuria strongly suggests haemorrhage at some point in the pathogenesis of CKD, but the authors did not comment (perhaps because they did not observe) whether the haemorrhage arose from capillaries, arteries or veins. In another relatively rare condition, Alport syndrome, a genetic abnormality in the expression of collagen IV leads to the dysfunction of the glomerular basement membrane, proteinuria and microscopic haematuria and, eventually, to inflammation and fibrosis, similar to the fibrosis seen in end-stage CKD [92]. In this study, too, the haemorrhaging vessels were not identified. Again, in polycystic degeneration of the kidney, also called polycystic CKD, haemorrhage within the cysts has been reported [93], although not the calibre of the vessels that bled. Thus, although these observations show that kidney haemorrhage seems to be reliably followed by fibrosis, more direct observation of vessels haemorrhaging in CKD has yet to be reported. These findings suggest the involvement of capillaries and invite further investigation.

### 2.2. Lung

In recent years, several studies have reported evidence of vascular involvement early in the pathogenesis of IPF. Lishnevsky and colleagues [94] have reported that capillary damage is an early event in a PECAM-deficient mouse model of lung fibrosis. In the human IPF lung, Puxeddo and colleagues [95] examined samples of epithelial lining fluid from sufferers of IPF and normal controls and reported exaggerated accumulation of iron in IPF broncho-alveolar ELF (alveolar epithelial lining fluid) and alveolar cells….. suggesting, occult pulmonary hemorrhage as a likely cause.

Ali and colleagues [96] confirmed the rise in iron accumulation in the IPF lung and suggested that the toxicity of iron might play a critical role in the pathogenesis of IPF. Their Figures 3B and 5A show Perls labelling of lung tissue from mouse models of IPF and from samples of human IPF lung, revealing iron-rich sites forming small spots, comparable to the ‘haem-rich deposits’ described in end-stage Alzheimer brains [11]. The spotty pattern suggests capillary haemorrhage, which was Cullen and colleagues’ interpretation, after considerable testing for the brain, but the point has still to be tested in the lung. Brown and Horvat [97] supported the idea that ‘dysregulated iron metabolism’ is causal in IPF, again without reference to the possibility that the abnormal iron levels observed might arise from haemorrhage.

The suspicion of ‘occult haemorrhage’, or at least blood vessel involvement, continued, however, in other studies. May and colleagues [98] reported that abnormalities of alveolar vessels are so marked that the endothelial cells (ECs) of these vessels, including capillaries, are likely involved, and suggested that EC cells are both causal and consequential in the pathogenesis of IPF. Sharma and colleagues [99] noted a correlation between the rate of fibrosis and the rate of transcription of vascular-related genes and suggested a key role of the vascular bed in the progression of IPF. Engelbrecht and colleagues [100] reached a similar conclusion from a survey of lung biopsies from IPF patients; they suggested that changes …. in the endothelium (of alveolar capillaries) may contribute to the development and/or progression of pulmonary fibrosis. Fliesser and colleagues [101] argued similarly that endothelial dysfunction—they discussed particularly capillary leakage—is central to the pathogenesis of IPF, but they also noted that it is well established by now that the pulmonary endothelium is affected in the course of lung fibrosis. However, to date, it is still not clear whether this is a cause or consequence of the fibrotic process.

Yang and colleagues [102], on the other hand, emphasised mechanical stress as a cause of alveolar fibrosis, although without considering the ageing pulse—a factor in our APICH hypothesis—as a source of stress. In addition, Sonaglioni and colleagues [103] noted that arterial elastance (a measure of the pressure the heart must generate to drive blood through the circulation) is correlated with the severity of IPF and suggested that the fibrosis that is affecting the lung may also affect the heart and the distributing arteries.

While these studies have given insight into the pathogenesis of IPF, none tested more directly whether the haemorrhage of alveolar capillaries occurs in human IPF. Put another way, the haemorrhage of cerebral capillaries has been well described in several forms of dementia and has been related to a prominent pathology of dementia (the senile plaque). In CKD and IPF, the investigation of capillary haemorrhage is a decade or two (if time is the right way to measure the gap) behind the comparable analysis of dementia. More haemorrhage-focussed investigation is needed to test the APICH hypothesis for the lung and the kidney.

## 3. What About the Liver? The Spleen? The Retina?

We have considered whether other highly vascular organs of the body should be grouped into the ‘triple mystery’ of the brain, kidney and lung.

The **liver** (blood supply: ~1 L/min) receives most its blood via a vein, the portal, which drains blood from the gut to the liver, whose roles include the metabolism of nutrients absorbed from food. Normal portal vein pressure is 5–10 mm Hg (https://en.wikipedia.org/wiki/Portal_hypertension (accessed on 1 July 2024)), well below the systemic arterial pressures to which the brain and kidney are exposed and half the normal pulmonary systolic pressure (~20 mm Hg) (Institute of Medicine (US) Committee on Social Security Cardiovascular Disability Criteria. Cardiovascular Disability: Updating the Social Security Listings. Washington (DC): National Academies Press (US); 2010. 11, Pulmonary Hypertension. Available from: https://www.ncbi.nlm.nih.gov/books/NBK209987/) (accessed on 1 July 2024). A minor part of the blood reaching the liver arrives via the hepatic artery, with typical systemic arterial pressure. This arterial blood mixes with the portal vein blood in the venous sinuses of the liver, apparently without complications. Even so, the liver is much less exposed to the pulse of the systemic circulation than the brain or kidney. A second distinct feature of the liver is that, uniquely among the body’s organs, it can regenerate functional tissue (https://www.nih.gov/news-events/nih-research-matters/cells-maintain-repair-liver-identified) (accessed on 1 July 2024). Perhaps for these reasons, there seem to be no idiopathic liver degenerations requiring explanation. Fatty liver degeneration is associated with obesity, diabetes and thyroid malfunction, and fibrotic degeneration (cirrhosis) with ingested toxins, like alcohol.

The **spleen** receives ~10% of the resting cardiac output (so ~0.5 L/min) for its several functions—the removal of red cells, the retrieval and recycling of iron from haemoglobin and pathogen detection in immune reactions. It differs from the brain, kidney and lung in that it is not essential to life and does not seem to degenerate with age. It can enlarge (splenomegaly), is vulnerable to external trauma to the torso and must be monitored after such trauma lest its capsule be torn, releasing blood into the peritoneal cavity. We could not find reports, however, of unexplained degenerations of the spleen. So, there are splenic mysteries, but they too are unlike the triple mystery that we argue is common to the brain, kidney and lung.

The many known **retinal degenerations** are best understood in two groups, those that affect the inner half of the retina (from the inner limiting membrane to the outer plexiform layer) and those that affect its outer layers, comprising the photoreceptors. The most common degenerations affecting the **inner** half of the retina are glaucoma, diabetic retinopathy and plaques. Glaucoma is a degeneration of ganglion cells, caused by the constriction of their axons as they pass through the lamina cribrosa [104]. This constriction is believed to be caused by a pressure gradient between the interior of the eye (normally ~15 mm Hg) and the cerebrospinal fluid (~7 mm Hg), which chronically tends to push retinal axons out of the eye, crushing them against the collagenous strands of the lamina cribrosa. Diabetic retinopathy is caused by glycation-induced breakdown of retinal vessels, which causes local hypoxia within the inner layers; and by a damaging neovascularisation induced by the hypoxia [105]. Recently, the formation of Aβ^+^ plaques in the inner retina of older patients in association with dementia has been described [106,107]. The retinal plaques are very similar to the senile plaques observed in the ageing brain, associated with Alzheimer’s dementia and their pathogenesis is likely to include APICH. That is, these plaques may be the same degeneration as seen in the cerebral cortex in several forms of dementia and, since the retina is an extension of the brain, this form of retinal degeneration is the same mystery as the dementias discussed above.

The degenerations of the outer retina are degenerations of photoreceptors, caused by mutations in genes specific to photoreceptor pigments, to enzymes of the phototransduction cascade or to proteins involved in the recycling of the discarded membranes of outer segments [108]. Because the outer retina is avascular (it is the only avascular region of the central nervous system), these degenerations cannot be caused by APICH. Again, the retina is a tissue with specialisations and mysteries, but its mysteries seem distinct from the triple mystery of our title.

## 4. Summary and Implications; Therapeutic Opportunity

This review began with the ‘mystery’ of the idiopathic failure of the three most vascular organs of the body. Here, we have proposed, and assembled evidence in support, that their degeneration may have a common cause in capillary haemorrhages caused by the ageing pulse—the APICH hypothesis set out above. Direct evidence that capillary haemorrhage is central to the pathogenesis of dementia, CKD and IPF is best developed for several dementias, and is summarised above. The direct evidence that vasculature is involved in the pathogenesis of CKD and IPF is substantial, but evidence of capillary haemorrhage, and of its causal role, is not yet available. The present evidence, we suggest, provides partial confirmation of our hypothesis and directs attention to the need for tests of capillary haemorrhage in both the kidney and lung.

### 4.1. Implications

The APICH hypothesis applied to the brain, kidney and lung provides new ways of thinking about dementia, CKD and IPF. The hypothesis suggests that the underlying pathology is a normal part of ageing, caused by a normal stress, the ageing pulse. The pulse-induced damage combines with lifestyle choices and genetic predispositions to determine the pace of the pathology, without offering a cure or even post-diagnosis treatment. It suggests that therapeutic hope lies in delay, avoiding the lifestyle issues that make the pulse more damaging, and pursuing ways, like acquired resilience [13], that can protect these organs.

A further implication is that perhaps the goal of a lifetime of providential lifestyle choices (things to do, including exercise, diet, weight control, exposure to photobiomodulation, intermittent fasting, regular saunas; things to avoid, including head-knocks, smoking, obesity) is the preservation of the organs’ reserves of functional tissue. It also provides a way of understanding why and when dementia, CKD and IPF are diagnosed—when tissue reserve is exhausted. Delay is neither prevention nor cure, but it can—the epidemiological data suggest—extend good health by a decade or three. The present analysis also suggests a reason why interventions commenced after diagnosis have such limited success—it is because tissue reserves are exhausted.

### 4.2. Conclusions

In 1992, Hachinski [109] advocated a proactive approach to the morbidities of vascular dementia, with measures like exercise, diet, cessation of smoking and antihypertensive drugs offered to patients considered at risk of strokes because they are elderly or suffering from atrial fibrillation, subtle cognitive impairment or silent cerebral infarctions. Three decades on, we would confirm, expand and qualify his insight. We suggest four expansions: (1) Because several dementias, including Alzheimer’s, are now increasingly understood as resulting from haemorrhage at the capillary level, these too should be regarded as vascular dementias and as equally preventable. (2) The kidney and lung—we argue above—suffer the same pulse-induced haemorrhage, so that CKD and IPF should be included with the dementias as preventable. (3) The preventive measures should be offered to the patient from mid-life when, the data discussed above suggest, the slow damage to brain, kidney and lung begins, well before symptoms of vulnerability to stroke appear. (4) The aim of the preventive measures is arguably better expressed as more than the prevention of strokes, rather as the preservation of the reserves of functional tissue in these organs that enable them to maintain high-level function as damage accumulates. The one qualification we would make to Hachinski’s view is as follows: (5) dementia, CKD and IPF are better understood as delayable rather than preventable, because the underlying capillary haemorrhage is caused by the ageing pulse. Everyone is threatened by these conditions, not just those who are already old and obviously at risk.

The concepts of delay and reserve help identify what we can—for hope—rely on and what is less likely to be effective, in particular the attempt to cure or reverse conditions after tissue reserves are exhausted and symptoms have appeared. These concepts suggest many testable, hypothesis-challenging questions.
